# Cost of Dengue Vector Control Activities in Malaysia

**DOI:** 10.4269/ajtmh.14-0667

**Published:** 2015-11-04

**Authors:** P. Raviwharmman Packierisamy, Chiu-Wan Ng, Maznah Dahlui, Jonathan Inbaraj, Venugopalan K. Balan, Yara A. Halasa, Donald S. Shepard

**Affiliations:** Julius Centre for Clinical Epidemiology and Evidence-Based Medicine, Department of Social and Preventive Medicine, Faculty of Medicine, University of Malaya, Kuala Lumpur, Malaysia; Selangor State Vector Borne Diseases Control Department, Selangor State Health Department, Ministry of Health, Selangor, Malaysia; Schneider Institutes for Health Policy, The Heller School, Brandeis University, Waltham, Massachusetts

## Abstract

Dengue fever, an arbovirus disease transmitted by *Aedes* mosquitoes, has recently spread rapidly, especially in the tropical countries of the Americas and Asia-Pacific regions. It is endemic in Malaysia, with an annual average of 37,937 reported dengue cases from 2007 to 2012. This study measured the overall economic impact of dengue in Malaysia, and estimated the costs of dengue prevention. In 2010, Malaysia spent US$73.5 million or 0.03% of the country's GDP on its National Dengue Vector Control Program. This spending represented US$1,591 per reported dengue case and US$2.68 per capita population. Most (92.2%) of this spending occurred in districts, primarily for fogging. A previous paper estimated the annual cost of dengue illness in the country at US$102.2 million. Thus, the inclusion of preventive activities increases the substantial estimated cost of dengue to US$175.7 million, or 72% above illness costs alone. If innovative technologies for dengue vector control prove efficacious, and a dengue vaccine was introduced, substantial existing spending could be rechanneled to fund them.

## Introduction

Dengue fever (DF), an arbovirus disease transmitted by the *Aedes* mosquito, has spread rapidly in the past six decades, and now 2.5 billion people, about 40% of the world's population, are at risk of infection. Estimates of global burden have varied. According to the latest estimates, annually about 390 million people are infected with 96 million apparent infections.^1^–^3^ Dengue is a disease of the tropics, and most cases occur in the countries of the Americas and the Asia-Pacific regions. With increasing disease burdens, dengue has exacted a high economic toll on the countries in these regions.^4^–^6^

**Figure 1.**
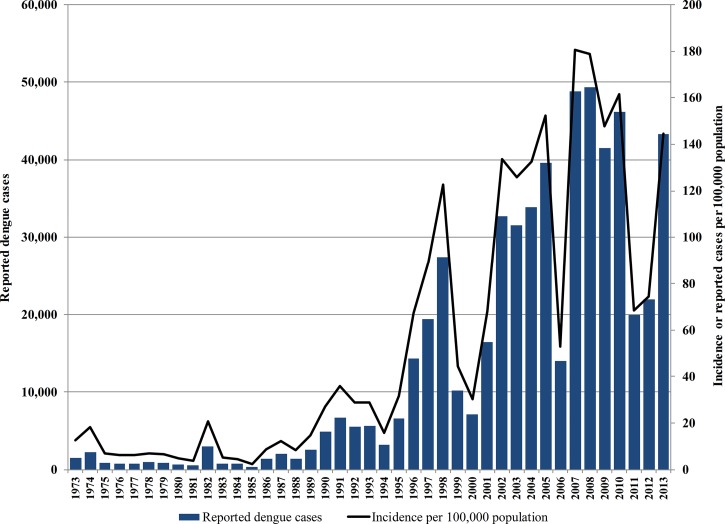
Trend of reported dengue cases in Malaysia, 1973–2013. Adapted from data obtained directly from the Vector Borne Diseases Control Sector, Disease Control Division, MoH, as well as other sources.^7^,^9^

Dengue is endemic in Malaysia, a tropical country of 27.5 million people located in southeast Asia. Cases of dengue were first described in the northern port city of Penang in 1902.^7^,^8^ Although there has been some presence of dengue in the country since then, incidence of reported dengue cases rose sharply beginning in the late 1980s (Figure 1

). Outbreaks of dengue tend to recur in 6-year cycles consisting of 4 years with high numbers of cases followed by 2 years with relatively low numbers. However, the annual average incidence of reported dengue cases in successive 6-year cycles has been increasing (Table 1). In the latest complete 6-year cycle, 2007–2012, dengue cases peaked from 2007 to 2010 with an annual average of 46,460 reported dengue cases (an incidence of 167 reported cases per 100,000 population) and 104 dengue deaths. In 2014, the cumulative number of cases and deaths in the first half of the year had outstripped these annual averages.^10^ The economic cost of dengue to the country is considerable. The cost of dengue illness, in terms of direct medical costs and costs related to productivity loss and premature mortality, amounted to US$102.2 million in 2009,^11^,^12^ the equivalent of 0.05% of the country's gross domestic product (GDP) for that year. However, these estimates still do not provide a complete picture of the national economic burden of dengue, as the costs of disease prevention had not been considered.

In Malaysia, dengue is a predominantly urban disease affecting mainly children and young adults aged between 10 and 30 years.^13^ The increase in dengue cases from the 1990s coincided with a period of rapid and, at times, less organized expansion of urban centers in the country. With crowded populations lacking reliable piped water, these areas could have contributed to high dengue transmission. As a primarily urban disease, dengue threatens vocal and politically influential urbanites in endemic countries. Industry and donors have been developing new control approaches. The first candidate vaccine has been successfully tested in southeast Asia and Latin America, and its developer was initiating regulatory filings in 2015.^14^,^15^ Innovative vector control measures are also being developed. Malaysia is at the forefront of pioneering genetically modified sterile insect techniques as a means of controlling the population of *Aedes* mosquitoes.^16^ The insertion of *Wolbachia* bacteria into mosquitoes is being tested in nearby countries.^17^

At present, however, prevention of dengue in the country must rely on existing integrated vector control approaches. These are aimed at reduction of mosquito breeding sites, environmental management, and the killing of adult and immature mosquitoes. In recognition of dengue's importance in Malaysia, the country's national dengue vector control program, led by the Ministry of Health (MoH), involves contributions from all levels of government—from the federal to the state and district level agencies.

Malaysia's passive dengue surveillance system, which began in 1973, requires health-care practitioners by law to report suspected cases of DF to the MoH within 24 hours of encountering such cases.^18^ Receipt of such reports triggers a chain of events aimed at containment of the disease. Vector control officers from the district health departments (DHDs) of the MoH receive notifications of clinically suspected dengue cases, investigate them, and initiate chemical fogging in areas surrounding the suspected index case as a means of destroying adult mosquitoes. DHD officers are assisted by vector control staff from the local authorities (LAs), public agencies involved in the public administration of large urban centers in the country. In addition to fogging, the DHDs intensify regularly occurring vector control activities, such as premise inspections for mosquito breeding sites and community education campaigns, to increase awareness of DF, its dangers, and methods of disease prevention. The MoH agencies at the federal and state levels are mainly responsible for providing technical support to the district level agencies, as well as maintenance of an effective regulatory framework for dengue vector control. The explicit annual budgets for the dengue vector control activities implemented by MoH agencies have been estimated to be US$17.8 million for the period 2002–2007.^19^ However, these budget allocations exclude costs borne by other public agencies. Thus, the full cost of Malaysia's dengue vector control program inputs and costs was not previously known.

This study aims to estimate the costs of the national dengue vector control program in Malaysia through examination of the inputs and costs incurred by public agencies at all levels of the government. The information was collected for the year 2010, a year of average incidence in the latest four outbreak years from 2007 to 2010. This information will enable Malaysia to join the handful of countries worldwide with a comprehensive estimate of dengue costs, combining costs for both illness and prevention. Such information will help gauge the overall economic impact of dengue in the country and inform the allocation of health resources.^15^,^20^ Data on dengue costs in Malaysia will help indicate how much money could potentially be reallocated to other control approaches as they become available.

## Methods

### Study area.

Malaysia is a federation of 13 states and three federal territories. In the administration of the country, the MoH, which is a federal government agency, is responsible for the overall governance of the health sector and provision of all levels of health care, from preventive to curative, throughout the country. The Federal Health Department (FHD), the highest level of the MoH, is supported by 14 state health departments (SHDs), which in turn are supported by 140 DHDs. Health matters in all three federal territories are jointly administered under one health department.

The three levels of the MoH (FHD, SHDs, and DHDs) have similar organizational structures, with units to administer major health programs replicated at each level of the MoH. However, departments at different levels of the MoH perform different functions. Thus, the Vector Borne Diseases Control Division of the FHD is responsible for macro-level administrative functions such as policy setting, program development, budget allocations, services, and facility planning. In addition to dengue, the vector-borne diseases program is also responsible for the control of malaria, chikungunya, Japanese encephalitis, plague, scrub typhus, yellow fever, and filariasis. However, because of the current low incidence or absence of these other conditions and increasing incidence of dengue in the country,^21^–^25^ this program focuses overwhelmingly on the control of dengue. The FHD oversees the functions of the Vector Borne Diseases Control Departments of the SHDs, which are responsible for the overall implementation of policies and programs related to vector-borne diseases in their states or federal territories. These departments are also involved in staff training and provision of technical advice to the Vector Borne Diseases Control Units of the DHDs. These are the departments directly responsible for delivering dengue vector control services, such as fogging and premise inspections, to the communities in each district.

Although, the MoH is overall in charge of health matters, certain local government agencies have also assumed responsibility to provide some supplementary preventive health services to their communities. All large towns in Malaysia have their own LAs, which are responsible for providing public services including public hygiene, sanitation, and vector control services. With more urbanized districts having multiple LAs, Malaysia has 151 LAs altogether. These agencies have established their own vector-borne disease control units. In the case of dengue prevention, the LAs deploy their control units primarily to assist DHDs in fogging activities triggered by reported dengue cases to the MoH. In some LAs, some fogging services are outsourced to private companies and performed under supervision of the respective LAs.

### Study sites.

Eight of Malaysia's 140 DHDs were selected for inclusion in this study. Sampling was done using the probability proportional to size (PPS) method^26^ based on the numbers of reported dengue cases in the districts for the year 2010. Since the intensity of dengue vector control activities in the districts is mainly related to the load of reported cases, sampling via PPS was chosen. This approach increased the likelihood of selecting districts with higher numbers of reported cases, but ensured that districts with few reported cases retained a chance for selection. Data for this study were collected from the vector-borne diseases control units of the eight selected DHDs and also the LAs in the same districts. However, there were multiple LAs in three of the eight selected districts: two LAs in each of two districts and three in the remaining district. In these districts, from all, a sole LA was randomly selected in the district using a simple random selection process.

The eight selected DHDs reported to four different SHDs. Of these, three SHDs were randomly selected to provide state-level dengue vector control costs for this study. Federal-level costs were collected from the sole Vector Borne Diseases Control Division of the FHD. Thus, the final list of 20 study sites included vector-borne diseases control units from eight LAs and 12 MoH sites. The MoH sites were made up of vector-borne diseases control units from eight DHDs, three SHDs, and the sole FHD. All these public agencies were invited to participate in this study and all agreed to do so. Table 2 provides details of the population sizes and numbers of reported dengue cases of the districts included in this study.

### Data on resource inputs and costs.

This study adopted a bottom-up costing approach^27^ in which all elements of the vector-borne diseases control program were first identified. Thereafter, data on resource utilization and unit costs of each resource were obtained. Information was collected to reflect resource use in the year 2010. The total program costs were then derived from the sum of the product of resource utilization and unit costs of each element.^28^,^29^ The perspective of the analysis is from the viewpoint of the funder, in this case the government, and thus only direct costs of the program have been included. Data collected included both capital and recurrent expenditures for dengue vector control activities. Capital items were assumed to have useful lifespans of 20 years in the case of buildings, 10 years for storage containers, and 5 years otherwise.^28^,^29^ All capital expenditures were annualized to capture apportionment of costs for 2010 using an annual discount rate of 3%.^30^ Where necessary, adjustments were made for inflation using consumer price indices obtained from the Department of Statistics, Malaysia.^31^

Data for resource use and costs at the district level were recorded in a matrix by line item and function. The seven line items, representing groupings of similar resources, are human resources (regular salaries and allowances, including overtime payments and wages of temporary workers hired during outbreaks), buildings, personal protective equipment (PPE), vehicles, fogging equipment, pesticides, and fogging services outsourced to private companies. All items combine amortized capital costs (e.g., buildings and equipment) and recurrent (e.g., utilities, fuel, and maintenance) costs. Thereafter, costs for the line items were summed up to provide the total cost of vector control activities for each district.

Similarly, the study identified five functional groups: premise inspection, entomological surveillance, fogging, larviciding, and health education. Unlike the breakdown of line items, disaggregation of costs by function was not obviously discernible. In each study site, a senior vector control officer (i.e., a person with more than 5 years' experience conducting vector control activities) provided the needed allocation based on the site's workload. Table 3 describes each line item and functional group for all levels.

Unlike activities at the DHDs, vector control activities at the SHDs and FHD are mainly administrative in nature and fell into only three line items: human resources, buildings, and vehicles. In addition, the FHD funds an annual mass media advertisement campaign aimed at increasing public awareness of the dangers of DF and encouraging public participation in vector control measures. Costs for this campaign were included as an additional line item for the federal department.

### Data collection.

A series of structured data collection forms, each detailing specific groupings of vector control resource inputs and costs, were developed for this study. A trained data collector approached personnel at all sites known to be in possession of data required for this study, namely officers from the vector control, human resources, engineering, and accounting departments. During the first encounter with each officer, the data collector explained in detail the specific vector resource inputs and costs required and asked the targeted staff members to complete the personnel data collection form. The data collector retrieved the completed forms in person a week after the initial meeting. If the officer was unable to provide the requested information at this time, the data collector provided further advice and assistance. If further information was needed, the data collector issued weekly reminders, either in person or via the telephone, until all the requested information had been given and the forms were completed and collected. Data collection was started in March 2012 and completed in August 2013.

If data on some category (salaries, vehicles, buildings, and fogging equipment) were still missing, the average unit or annual costs for similar resources obtained from other study sites were used as a proxy. Overall, about 11% of cost components had some incomplete data, but reasonable estimates could be generated from related quantities. For example, sometimes an LA could not supply information on the salary of a health inspector but knew the number of such officers. The cost was then estimated by multiplying the average salary of a health inspector from other sites with the number of health inspectors at that LA. Often, data were missing for only one part of the component, so actual data could be used for the other parts of that component.

### Estimation of district vector control costs.

The total vector control costs in each of the eight sampled districts were derived by combining the vector control costs for the DHD and LA in that district. However, in three of the sampled districts, there was more than one LA. In each of these districts, data on resource use and costs were collected from only one LA, selected randomly from all the LAs in the district. Data on the number of reported dengue cases were collected for all the LAs, both selected and nonselected. In each sampled LA, vector control costs for each line item were broken down per reported case. As the vector control activities in LAs were implemented in response to reported cases, these estimates per reported case were then applied to the number of reported cases in the non-sampled LAs to generate overall costs of LAs by line item in each district. Finally, we derived district-level costs and breakdowns by line item and function, by combining the costs of LAs with those of DHDs.

### Estimation of national vector control costs.

The vector control costs collected from the district, state, and federal levels were then used to estimate the national costs for dengue vector control in Malaysia. The costs for each sampled district were inflated (multiplied) using sampling weights, which were the inverse of the probability of each district being sampled.^32^ The sum of the inflated district costs provided the estimated vector control costs for all districts in Malaysia. Unlike vector control activities at the level of the district, dengue control activities provided by the SHDs are not wholly dependent on the number of reported cases or population size in each state. Moreover, there was little variation in the staffing among SHDs. Thus, in this study, the average vector control costs per SHD estimated from the three sampled SHDs were multiplied by 14 to generate the total costs for all SHDs in the country. Finally, the estimates of vector control costs for the district, state, and the federal levels were summed to provide the estimated national dengue vector control costs for Malaysia for the year 2010.

### Data analysis.

Data were analyzed using Excel (Microsoft Corp., Redmond, WA) and the statistical package IBM SPSS Statistics version 21 (IBM Corp., Armonk, NY). First, cost estimates were reported by total vector control costs, costs per reported dengue case, and costs per capita at the district, state, federal, and finally national levels. Then, to generate 95% confidence intervals (CIs), district- and state-level costs were bootstrapped with 10,000 repetitions. All costs are reported in U.S. dollars (US$) using the average 2010 exchange rate of US$1 equals to 3.20 Malaysian ringgit.

### Ethics statement.

This study was registered with the National Medical Research Registry of Malaysia (registration no. NMRR-11-263-9217) and approved by its Ethics Committee.

## Results

### District-level vector control costs.

In 2010, there were 46,171 reported dengue cases throughout Malaysia. Of this total, 16,676 cases or 36.1% were reported in the eight diverse districts included in this study (Table 2). Among these study districts were three districts with the highest dengue burden in the country, namely Petaling, Hulu Langat, and Gombak, which in 2010 had 13,106 reported dengue cases, or 28.4% of the cases reported for the entire country. This study also included the district of Sik with 71 reported cases, which was one of the lowest dengue burden districts in the country.

Table 2 shows that at the level of the districts, dengue vector control costs ranged from US$0.2 million in Sik to US$2.8 million in Gombak. The average district vector control cost was US$1.4 million. A linear regression confirmed, as expected, that DHDs with more annual reported dengue cases (Cases) tended to have more costly vector control expenditures. The resulting regression equation was as follows: DHD cost (in US$) = $622,000 + Cases × $380 (*R*^2^ = 0.790, *N* = 8, *P* = 0.019). In general, the cost per reported case appeared to be lower in districts with higher burden of cases compared with those with lower burdens. The average cost per reported case at the district level was US$1,467. The main cost drivers in the districts were for human resource and pesticides. On average, 60.7% of dengue vector control costs in the districts were for human resources and 13.6% of the costs were for pesticides. There were 826 government staff contributing to vector control efforts in the eight districts. However, not all of them were involved full-time in dengue vector control. There were only 685 full-time equivalent (FTE) staff dedicated to dengue-related activities. Thus, the average number of staff in each district was 103 persons or 86 FTE staff (Table 4). About 89.6% of the staff were health-care professionals or persons who had received specific training for dengue vector control activities such as doctors, entomologists, and allied health professionals such as health inspectors. The remainder of the staff, which included clerks, drivers, and cleaners, provided administrative support. Pesticides used for fogging and larviciding activities included both liquid and powder forms. On average, each district used 6,774 L of liquid-based pesticides and 590 kg of powder-based pesticides. A summary of the resources used for vector control activities in the districts is provided in Table 4.

### State and federal dengue vector control costs.

Unlike district-level costs, vector-control costs incurred at the state level showed less variation, ranging from US$0.2 million in the state of Malacca to US$0.3 million in Kedah (Table 2). The average state vector control cost was US$0.3 million. At the federal level, the estimated vector control costs came to US$1.7 million. Since vector control activities at the FHD and SHDs are mainly administrative in nature, the main cost driver at these levels was for human resources.

### National dengue vector control costs.

**Figure 2.**
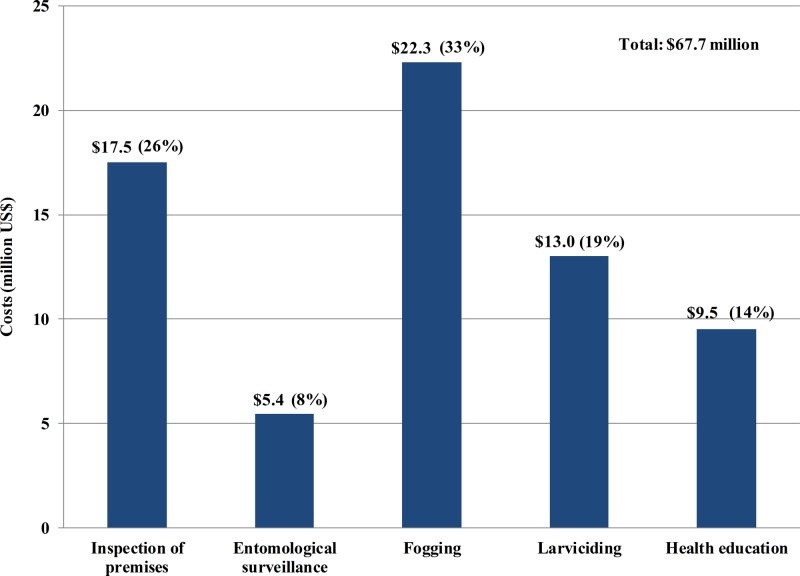
District-level dengue vector control costs by functional group, Malaysia, 2010.

In 2010, Malaysia spent an estimated US$73.5 million (95% CI = US$62.0–US$86.3 million) on the national dengue vector control program (Table 5). The estimated costs per reported dengue case and per capita population were US$1,591 (95% CI = US$1,343–US$1,870) and US$2.68 (95% CI = US$2.26–US$3.15), respectively. About 92.2% of these costs were incurred at the district level, where most of the costs went to fogging activities and premise inspection for mosquito breeding sites (Figure 2

).

Overall, 91.4% of the total national costs for dengue vector control activities were for recurrent expenditures, mainly for payment of the salaries and allowances of the health-care personnel involved either directly or indirectly in these activities. Human resource costs made up 64.8% of total national vector control costs. The cost of pesticides themselves amounted to only 10.9% of the total costs. However, the application of pesticides also entailed human resource, vehicles, equipment, and other inputs listed separately in Table 5.

## Discussion

DF is the most important vector-borne disease in Malaysia in terms of disease and economic burdens. The 46,171 dengue cases and 134 dengue deaths reported in the country in 2010 are, by far, much higher than those for other known vector-borne diseases in Malaysia, such as malaria (6,650 cases and 33 deaths)^33^ and chikungunya (804 cases with no recorded deaths).^34^ Partially in response to public demands for action, the government has invested substantially in the national dengue prevention program. We estimate that US$73.5 million in public funds was spent on dengue vector control activities in 2010. This figure constitutes 0.03% of the country's GDP of US$247.5 billion in 2010^35^ and 1.2% of the total government funding for health care in Malaysia of US$6.0 billion.^36^

Malaysia's financial commitment to dengue prevention is not unique in the region. Other southeast Asian countries facing significant dengue burdens have also invested substantially in dengue prevention activities. Cambodia spent an average of US$567,800 annually for the period 2001–2005 on larviciding campaigns to prevent dengue in densely populated areas, mainly in the capital city of Phnom Penh and its adjoining province Kandal, areas that covered only 23% of the country's population.^37^ The Singapore government spent US$50 million for dengue control for the entire island nation in 2010.^38^ Thailand's public sector dengue vector control costs, including health education and larviciding came to US$62 million in 2005.^39^ Using country-specific current GDP estimates for the relevant years available from the World Bank database,^35^ dengue control costs reported in these studies would amount to an average of 0.01% of the GDP of Cambodia for the period 2001–2005, 0.02% of the GDP of Singapore in 2010, and 0.04% of the GDP in Thailand for 2005.

Despite the substantial investment in dengue vector control, the effectiveness of the national program has yet to be fully evaluated. At the level of the districts, vector control activities were conducted mainly to prevent transmission of dengue from suspected cases, rather than to eliminate the disease. This approach may be driven by public expectations of government reaction to dengue outbreaks in the country rather than higher expectations of proactive actions to prevent dengue outbreaks. This is reflected by our finding that one-third of the costs for dengue prevention activities were incurred for killing of adult mosquitoes through chemical fogging. However, there are several concerns related to use of insecticides in dengue prevention, namely the development of mosquito resistance, environmental risks, and the transient variable efficacy of peridomestic space spraying.^40^–^44^ Further research is required in Malaysia to determine the impact of insecticide use in dengue vector control.

Dengue vector control activities as practiced in Malaysia are intensely human resource dependent. A large human workforce is required to perform the variety of dengue vector control, surveillance, and prevention activities at the district level. Trained allied health professionals conduct premise inspections, fogging and larviciding activities, the mainstay of the national vector control program. Other public health professionals, including doctors and entomologists, provide technical support. As a consequence, human resources' costs contribute the largest portion of the overall program costs.

In contrast to other aspects of dengue vector control, only 15% of the total cost of the national program was spent on health education and promotion efforts, inclusive of activities in the districts and the national mass media advertisement campaign. This is approximately three times less than the costs of fogging and larviciding activities. Social mobilization and communication are important measures to ensure sustainable dengue prevention and control activities.^45^–^47^ Educational messages embedded in a community-based approach have an important impact on reducing larval indices, as opposed to fogging activities that only target adult mosquitoes.^9^

The major strength of this study comes from the use of micro-costing to estimate dengue vector control costs through a bottom-up approach in which all vector control resources were identified. We collected inputs and cost data directly from the vector control units from a representative selection of public agencies. We believe this study is the first attempt to use systematic and comprehensive cost methods to estimate dengue vector control and prevention in Malaysia.

There are several limitations to this study. Our cost perspective is limited to the public sector only; we did not include the cost of dengue vector activities paid for by private corporations (e.g., fogging activities surrounding hotels, factories, and warehouses) and private households (e.g., fogging conducted in areas surrounding private condominiums and residential apartments). These services are generally conducted at regular intervals by private pest control companies. Although such services are primarily aimed at prevention of DF, they are only carried out in a small proportion of workplaces and residential apartments. We also did not include the private household expenditures for purchase of mosquito coils, insecticide spray cans, and window mosquito nettings. However, these items are mainly used to deter nuisance mosquitoes rather than prevention of DF. We have also not included community mosquito prevention activities conducted by nongovernmental organizations, which are mostly conducted on an ad hoc basis. On the other hand, our estimate of costs of pesticides may have included some products used against *Aedes albopictus* for chikungunya control. However, this amount would have been small, as reported chikungunya cases were fewer than 2% of the reported dengue cases.

In summary, Malaysia is an upper-middle-income country that spends annually approximately 5% of total GDP on health overall and 0.03% specifically on dengue vector control. Dengue poses a significant economic burden to the country, with a combined annual cost for prevention and illness of US$175.7 million. Malaysia has been reliant on a government-funded integrated dengue vector control program, which includes efforts to garner community support through health education activities. These approaches have not been able to prevent dengue outbreaks in the country. Innovative control technologies against this disease include the *Toxorhynchites* larvae (a biological control method for the dengue vector),^48^ genetically modified sterile mosquitoes, *Wolbachia* inserted into mosquitoes,^17^ and the dengue vaccine.^14^ This study's quantification of the disease's economic burden informs policy makers and stakeholders regarding the implementation of existing and new technologies for controlling dengue.

## Figures and Tables

**Table 1:** Reported dengue cases and dengue deaths during successive 6-year cycles from 1989 to 2012, Malaysia

Period	Average annual no. of reported dengue cases	Average incidence of reported dengue cases per 100,000 population	Average annual number of dengue deaths
1989–1994	4,716	25.0	22.7
1995–2000	14,143	64.2	40.7
2001–2006	28,033	110.9	80.3
2007–2012	37,937	135.2	81.2

Adapted from data obtained directly from the Vector Borne Diseases Control Sector, Disease Control Division, MoH, as well as other sources.^7^,^9^

**Table 2:** Characteristics and dengue vector control costs at district, state, and federal levels (2010)

Study site	Population (million)	Reported dengue cases	Total dengue vector control costs (US$million)	Dengue vector control costs per reported case (US$)	Dengue vector control costs per capita population (US$)
Districts					
Gombak*	0.7	3,107	2.85	915.94	4.26
Petaling*	1.8	5,147	2.75	534.34	1.56
Hulu Langat*	1.1	4,852	1.53	314.48	1.34
Klang*	0.8	1,752	1.42	810.96	1.69
Melaka Tengah†	0.5	1,048	1.39	1,325.64	2.87
Batu Pahat‡	0.4	175	0.75	4,289.24	1.87
Kuala Langat*	0.2	524	0.43	814.61	1.94
Sik§	0.1	71	0.19	2,730.34	2.92
Mean (SE)	0.7 (0.2)	2,085 (775)	1.41 (0.35)	1,466.94 (480.68)	2.30 (0.35)
States					
Selangor	5.3	16,367	0.29	17.97	0.06
Malacca	1.9	1,485	0.22	149.49	0.28
Kedah	0.8	782	0.34	436.66	0.18
Mean (SE)	2.7 (1.4)	8,802 (5,081)	0.29 (0.03)	201.37 (123.62)	0.17 (0.07)
Federal					
Federal	27.5	46,171	1.72	37.21	0.06

SE = standard error.

* Districts in the state of Selangor.

† District in the state of Malacca.

‡ District in the state of Johore.

§ District in the state of Kedah.

**Table 3:** Description of line items and functional groups

Category	Description
Line items	
Human resources	Annual salaries and other allowances for staff such as overtime claims, housing and uniform allowances, and wages for temporary workers hired during outbreaks
Buildings	Buildings used for administration of programs as well as for storage of equipment and is inclusive of both capital (annualized purchase price or annual rentals) and recurrent costs (e.g., insurance, utilities, and maintenance)
Vehicles	Vehicles used in vector control activities such as fogging activities and is inclusive of both capital (annualized purchase price or annual rental) and recurrent costs (e.g., fuel, maintenance, and insurance)
Fogging equipment	Fogging/larviciding equipment, either ULV equipment mounted on pick-up trucks or thermal fogging machines carried on the back of vector control officers, and is inclusive of both capital costs (annualized purchase price) and recurrent costs (fuel and maintenance)
Pesticides	Insecticides used for larviciding and fogging activities
PPE	PPE including goggles, mask, gloves, respirator, boots, and so on, used during larviciding and fogging activities
Outsourced services	Costs of fogging and larviciding activities subcontracted to private companies
National dengue prevention advertisement campaign*	Costs of national broadcasting in radio, television, and local newspapers, including hiring of celebrities to promote dengue prevention campaigns
Functional groups	
Inspection of premises	Inspection of buildings including houses, shops, construction sites, and schools for mosquito breeding sites
Entomological surveillance	Activities to collect data for entomological indices, such as *Aedes* and Breteau indices
Fogging	Back-mounted thermal fogging and truck-mounted ULV fogging at premises and areas found to have dengue cases
Larviciding	Application of insecticides at potential breeding sites of premises and areas found to have dengue cases
Health education	Activities to educate the community including distributing flyers, pamphlets, brochures, giving educational talks, banners and buntings, and engaging local community leaders through the COMBI programs to spearhead campaigns to keep the living environment clean and mosquito free

COMBI = Communication for Behavioral Impact; PPE = personal protective equipment; ULV = ultra-low volume.

* This line item applies only at the Federal Health Department.

**Table 4:** Selected resources used for dengue vector control in study districts

Category of resource	Mean	Standard error	Minimum	Maximum
Total number of staff	103	22	25	198
% Professional* staff	89.6	–	–	–
% Administrative† staff	10.4	–	–	–
FTE for dengue vector control	86	19	17	176
Pesticides				
Liquid-based pesticides (L)	6,744	2,721	312	24,814
Powder-based pesticides (kg)	590	195	2	1,597
Diesel‡ (L)	106,431	64,721	4,972	551,266
Fogging and larviciding equipment	52	9	21	90
Annual servicing and maintenance costs (US$)	20,526	7,551	1,797	66,373
Vehicles	15	4	4	38
% Dedicated to dengue vector control	87.6	–	–	–
% More than 5 years old	39.2	–	–	–
Annual servicing and maintenance costs (US$)	16,151	3,736	3,473	36,625

FTE = full-time equivalent.

* Professional staff are persons trained for dengue vector control, surveillance, and prevention activities, and they include doctors, entomologists, and health inspectors.

† Administrative staff are persons performing administrative or general duties such as clerks, drivers, cleaners, and so on.

‡ Refers to diesel used to dilute oil-based pesticides for fogging activities.

**Table 5:** Dengue vector control costs by line item and level of government, Malaysia, 2010*

Line item and totals	District level	State level	Federal level	All levels
Aggregate (US$million)				
Human resource	44.41 (38.43–50.92)	3.06 (2.11–3.98)	0.14	47.61 (42.55–55.03)
Building	3.87 (2.99–4.97)	0.65 (0.55–0.74)	0.04	4.56 (3.78–5.76)
Vehicles	5.15 (4.17–6.42)	0.29 (0.25–0.35)	0.01	5.44 (4.53–6.77)
Fogging equipment	3.89 (2.80–5.31)	NA	NA	3.89 (2.80–5.31)
Pesticides	8.02 (5.97–10.57)	NA	NA	8.02 (5.97–10.57)
PPE	1.83 (1.62–2.06)	NA	NA	1.83 (1.62–2.06)
Outsourced fogging services	0.57 (0.00–1.29)	NA	NA	0.57 (0.00–1.29)
National dengue prevention advertisement program	NA	NA	NA	1.53
Total (US$million)	67.73 (57.20–79.85)	4.00 (3.11–4.78)	1.72	73.45 (62–86)
Per reported case (US$)	1,467.02 (1,239–1,729)	86.6 (67.31–103.54)	37.21	1,590.90 (1,343–1,870)
Per capita population (US$)	2.47 (2.09–2.91)	0.15 (0.11–0.17)	0.06	2.68 (2.26–3.15)

NA = not applicable; PPE = personal protective equipment.

* Numbers in parentheses are bootstrapped 95% confidence interval values.
